# Commentary: “Hearing, touching, and multisensory integration during mate choice” – Sex, Drugs and Leather Jackets

**DOI:** 10.3389/fncir.2022.1080276

**Published:** 2022-12-08

**Authors:** Gonzalo R. Quintana

**Affiliations:** Departamento de Psicología y Filosofía, Facultad de Ciencias Sociales, Universidad de Tarapacá, Arica, Chile

**Keywords:** partner preference, conditioned ejaculatory preference, mate choice, somatosensory, fetish, opioid, ventral tegmental area (VTA), medial preoptic area (mPOA)

## Introduction

Lenschow et al. (2022) reviewed the recent findings related to the literature on hearing and touching in partner/mate choice/preference in animal models, as well as the multisensory integration circuits in this phenomenon. Their review covered evidence from rats, mice, and humans, while distinguishing groups by sex and evaluating the contribution of vocalizations and somatization during premating, mating, and post-mating choice, to further analyze the synergistic action of multisensory cues in this phenomenon.

The authors carried out great work describing, organizing, and discussing the available research, providing a wide breadth of the literature. They also acknowledge being unable to include all relevant studies due to space constraints. Therefore, in light of this limitation, the following commentary will provide essential missing pieces of evidence regarding the manipulation of somatosensory cues in partner/mate preference/choice, and will discuss the role of opioids in this phenomenon.

## Animal evidence on somatosensory cues in partner/mate preference/choice

Domjan et al. were perhaps the first to demonstrate experimentally that animals can develop sexual fetishes. They trained Japanese quail males to associate an inanimate taxidermy quail female before copulation with a sexually receptive partner (SRP). In the “fading” group, the taxidermic female was gradually covered with terrycloth, to be fully covered leaving no quail features at the last trial of training. In the “non-fading” group, the taxidermic female was always presented fully covered. At the test session, males were presented only with the fully covered taxidermic female. Males in the fading group spent more time around the taxidermic female and displayed more copulatory behaviors toward it (e.g., grabs, mounts, and cloacal contacts) than those in the non-fading group (Domjan et al., 1992). They also showed persistence of copulation with a taxidermic female even after 20 trials of extinction, but this was not reflected when using light as a conditioned stimulus, nor when food was used as an unconditional stimulus instead of an SRP female quail (Köksal et al., 2004). Interestingly, considering that an important part of partner/mate preference/choice is undeniably related to reproduction, when given the opportunity to copulate with a real SRP, quail males who copulated with the taxidermic female fertilized a greater proportion of eggs than control males (Cetinkaya and Domjan, 2006).

Quintana et al., moreover, demonstrated that a somatosensory cue can be used as a conditioned stimulus to drive partner/mate preference/choice in male and female rats. Quintana et al. (2019b) found that animals were able to copulate with SRP wearing a rodent jacket for 14 training trials and they were later tested in an open field for their preference, using two random SRP, one with the jacket on and the other with the jacket off. Males who copulated with females wearing jackets showed a preference toward jacketed partners, whereas females only showed a mate/partner preference/choice when the jacket was associated with SRP during the training, and its absence indicated sexually non-receptive partners. Previous research has consistently demonstrated that these rewarding associations are mainly the product of opioid transmission in several brain areas (Pfaus et al., 2012). Thus, a follow-up study (Quintana et al., 2019c) used a similar training strategy, but male rats were either injected with naloxone, an opioid antagonist, or a saline solution before each trial. During the same preference test, while injecting saline into both groups, they found that males injected with saline during training displayed a preference toward jacketed females, whereas males injected with naloxone displayed a preference for unjacketed females. Among the many brain areas where opioids are known to facilitate a conditioned partner/mate preference/choice, two major brain hubs are the medial preoptic (mPOA) and ventral tegmental areas (VTAs; Pfaus, 2009; Georgiadis et al., 2012). Quintana et al. (2019a) found that when they microinjected naloxone into the mPOA of males trained to associate the jacket with SRP, their partner/mate preference/choice shifted toward unjacketed females, whereas microinjections of naloxone into the VTA only abolished that preference. Subsequent detection of c-Fos protein induced by the jacket showed that, relative to the control group, microinjections of naloxone into the mPOA suppressed c-Fos in both the mPOA and VTA, whereas microinjections of naloxone into the VTA suppressed c-Fos only in the VTA. These findings demonstrate that for a partner/mate preference/choice, a somatosensory cue works as a conditioned stimulus just like when using cues from other sensory modalities (e.g., olfactory; Pfaus et al., 2012), likely through similar neural pathways.

It has also been shown that a somatosensory cue can modulate sexual arousal and behaviors in those who wear a fetish cue. Pfaus et al. (2013) gave males, either wearing or not wearing a jacket, several copulatory experiences with SRP. During the test, half of the males wore the jacket, whereas the others did not. Those who did not wear a jacket during training or the test copulated normally, as did those who wore a jacket during training and the test, and those who did not wear the jacket during training but were tested with it. However, males who wore the jacket during training but not during the test made significantly fewer anticipatory level changes, had fewer longer mount, intromission, ejaculatory latencies, and ejaculated significantly less. Similar results were found when males were trained to associate wearing the jacket with sexually non-receptive females, and then tested while wearing the jacket.

## Discussion

While it is undeniable that animals copulate to reproduce, there have been numerous reports of animals of different species copulating with different objects, fetishes, or not-sexual targets (Young, 1949; Beach, 1950; Barraud, 1953; Ficken and Dilger, 1960). Indeed, partner/mate preference/choice is known to be fostered through opioid transmission responsible for sexual pleasure and reward, which in turn sensitizes dopamine, oxytocin, and vasopressin systems responsible for attention, arousal, and bonding, along with the learned experience that ultimately determines not only who but also what is sexually attractive and arousing (Quintana et al., 2022). The same mechanisms are also used to predict why humans deviate from the reproductive aspects of this phenomenon (Pfaus et al., 2020).

## Author contributions

The author confirms being the sole contributor of this work and has approved it for publication.
